# Elevated serum antibody against *Schistosoma japonicum* HSP60 as a promising biomarker for liver pathology in schistosomiasis

**DOI:** 10.1038/s41598-017-08283-5

**Published:** 2017-08-10

**Authors:** Xiaojun Chen, Wei Li, Yalin Li, Lei Xu, Sha Zhou, Jifeng Zhu, Zhipeng Xu, Feng Liu, Dandan Lin, Fei Hu, Yuemin Liu, Wen Jiang, Liwang Cui, Chuan Su

**Affiliations:** 1 Department of Pathogen Biology & Immunology, Jiangsu Key Laboratory of Pathogen Biology, Nanjing Medical University, Nanjing, Jiangsu 210029 P. R. China; 2 Jiangxi Provincial Institute of Parasitic Diseases, Nanchang, Jiangxi 330046 P. R. China; 3 Department of Biochemistry and Molecular Biology, Nanjing Medical University, Nanjing, Jiangsu 210029 P. R. China; 4 Department of Entomology, Pennsylvania State University, University Park, Pennsylvania 16802 USA

## Abstract

The pathology associated with *Schistosoma japonicum* (*S. japonicum*) infection in humans is attributed to parasite egg-induced granulomatous inflammation and fibrosis in the host liver. Currently, a marker that is reliable, cheap, less device-dependent, and can be easily and repeatedly used on a large scale to monitor the progression of liver pathology in schistosomiasis japonica endemic areas is lacking. The levels of serum *S. japonicum* heat shock protein 60 (SjHSP60)-specific IgG and its subtype antibodies in animals (mice and rabbits) or patients with schistosomiasis were measured by ELISA. Liver pathologies in mice and rabbits were evaluated by gross pathology and histopathology, and hepatic fibrosis in patients was examined with ultrasound imaging. The results revealed that the titers of the total IgG and subtype IgG1 anti-SjHSP60 antibodies were positively correlated with the severity of liver pathology after *S. japonicum* infection. Our findings indicate that the SjHSP60 IgG and IgG1 antibody levels can be used as potential candidate biomarkers for evaluation of liver pathology in schistosomiasis; however, validation remains to be explored in further work.

## Introduction

Schistosomiasis remains a major public health problem worldwide and is endemic mainly in the poor and undeveloped countries or areas^1^. Among three major pathogenic schistosome species, *S. japonicum* is responsible for human and animal infections in parts of East and Southeast Asia, primarily China, the Philippines and Indonesia^2, 3^. It is well established that *S. japonicum* infection causes granulomatous responses to parasite eggs trapped in the liver, which subsequently results in serious liver fibrosis and circulatory impairment and eventually leads to the loss of the ability to work and self-care and even the death in the patients^4^. Thus, the dynamic evaluation of liver pathology is fundamental for implementing appropriate therapeutic interventions in liver fibrosis in schistosomiasis patients. However, there are still few efficacious approaches that are reliable, cheap, less device-dependent and can be easily and repeatedly used to monitor liver pathology on a mass scale particularly in patients in poor or remote endemic areas.

Schistosome egg-triggered granuloma and pipestem fibrosis are located within the liver sinusoids and the branches of the portal vein and affect the interspaces of hepatocytes rather than the hepatocytes^5, 6^. Accordingly, the hepatocytes are preserved, and the structure and functions of the liver are less affected during both the acute and chronic stages of schistosomiasis^7^. Thus, the levels of liver enzymes, such as AST and ALT, are less affected in schistosomiasis patients until the very late stage, when the hepatocytes are damaged from serious blockage of blood flow by extreme over-development of pipestem fibroses^8^. Therefore, the levels of liver enzymes, although easy and relatively cheap to access, cannot be used to monitor the progression of liver pathology in schistosomiasis patients. Instead, expensive and/or high-tech device-reliant methods, such as computed tomography (CT), magnetic resonance imaging (MRI), and especially ultrasonography are commonly used to assess the severity of liver damage in schistosomiasis. However, the screening sensitivity of these methods is under discussion, particularly during mild hepatic damage^9, 10^. Additionally, these methods are time-consuming, high-tech device-dependent, expensive, and may not be suitable for repeated use in mass-screen patients or for epidemiologically monitoring the progression of liver pathology, especially in the poor and remote endemic areas. Therefore, sensitive, simple, less device-dependent and affordable screening and monitoring tests are urgently needed for liver pathology progression in schistosomiasis.

Antibody responses to the heat shock protein 60 (HSP60) from various pathogens have been used as a proxy for the pathology during infection because the severity of infection is difficult to accurately evaluate with conventional and non-invasive detection methods^11^. For example, substantial evidence supports the notion that *Chlamydial* HSP60 is certainly a clear immunopathology-associated antigen, and the titer of anti-*Chlamydial* HSP60 antibody is associated with disease pathology^12–15^. *Helicobacter pylori* HSP60 IgG antibodies are correlated with the grade of chronic inflammation in the gastric mucosa^16^. Previous studies have provided substantial evidence that elevated levels of *Mycobacterial* HSP60/65 antibodies are significantly associated with carotid artery thickening and coronary calcification levels^17–19^. Our previous study demonstrated that SjHSP60 is constitutively expressed in both the eggs and adults of *S. japonicum* ^20^, which agrees with the above published reports and raises the possibility that antibodies against SjHSP60 might serve as a biomarker for monitoring the progression of pathology in schistosomiasis japonica.

In this study, we investigated whether SjHSP60 antibodies could serve as a biomarker and are associated with the severity of liver pathology in hosts with schistosome infection. In both infected animals and human patients, we found that the levels of SjHSP60 antibodies were significantly correlated with the severity of liver pathology, which suggests that SjHSP60 antibody could be a biomarker for the screening and monitoring of liver pathology in schistosomiasis japonica.

## Results

### Correlation between the SjHSP60 antibody level and the severity of liver pathology in the acute stage in mice

**Figure 1:**
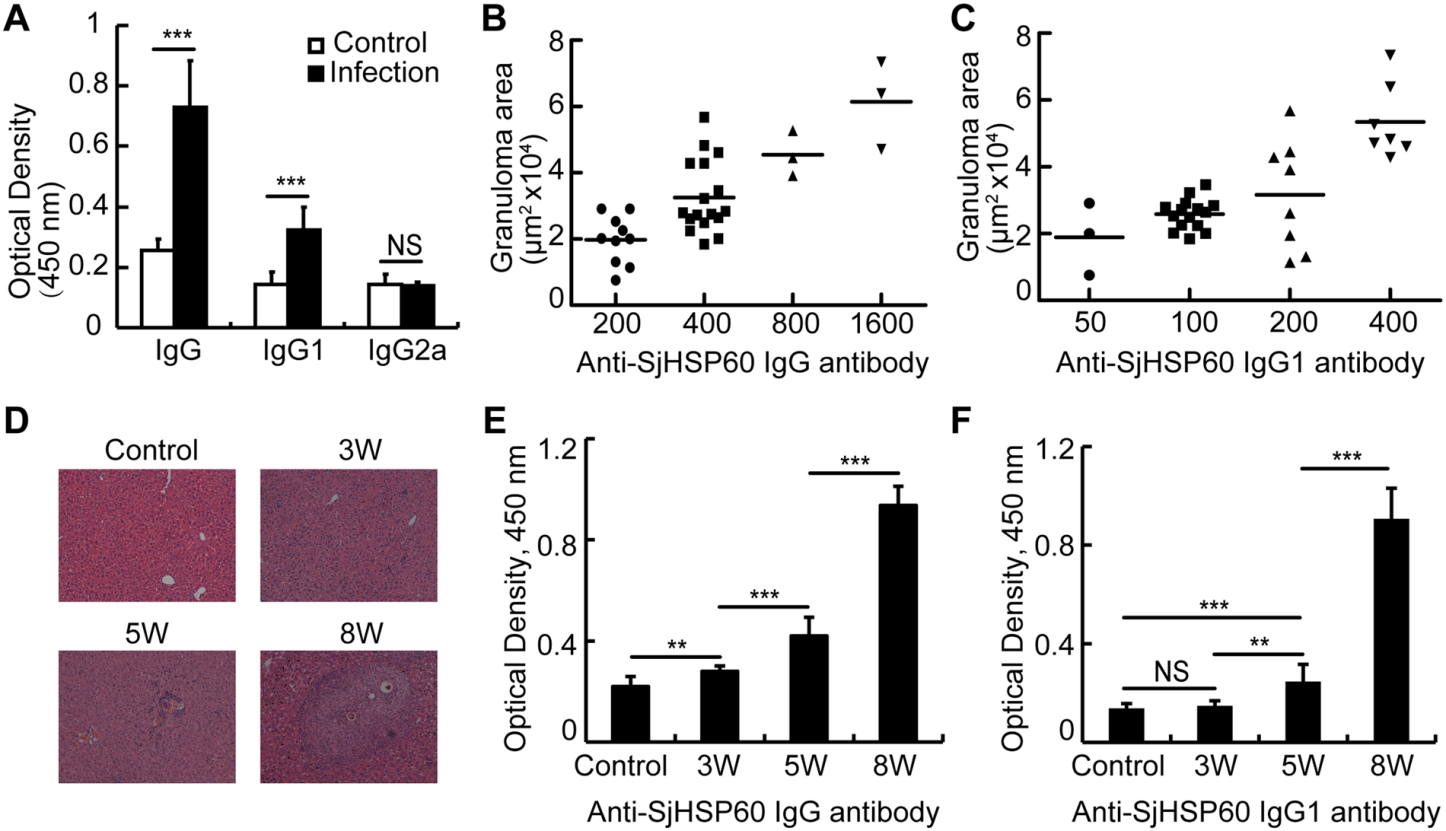
Correlation between the levels of SjHSP60 antibody and the sizes of the hepatic granulomas in the *Schistosoma japonicum*-infected mice. (**A**) C57BL/6 J mice were infected with or without 12 *S. japonicum* cercariae as described in the Materials and methods. Sera were obtained 8 weeks after infection. Anti-SjHSP60 total IgG, IgG1, or IgG2a antibodies were determined by ELISA in triplicate wells. The results are presented as the means ± the SDs from six mice in each group and are representative of 3 independent experiments. ***P < 0.001, NS indicates not significant (Student’s *t*-test). (**B,C**) C57BL/6 J mice were infected with or without 12, 20 or 40 *S. japonicum* cercariae. Eight weeks later, sera and liver slices were harvested. Associations between the titers of SjHSP60 antibodies and the sizes of the granulomas in the *S. japonicum*-infected mice were analyzed by Spearman’s rank correlation. The sizes of granulomas around single eggs were quantified with the AxioVision Rel 4.7. The data are expressed in area units (n = 33). The ELISA data are expressed in serum dilution (1/x). The data are representative of 3 independent experiments. (**D,F**) C57BL/6 J mice were infected with 12 cercariae of *S. japonicum* per mouse. Three mice were randomly chosen and sacrificed at 0 (before infection), 3, 5 or 8 weeks post-infection. The data are representative of 3 independent experiments. Histopathology in the livers (**D**). Anti-SjHSP60 total IgG (**E**) and IgG1 (**F**) antibodies were determined by ELISA in triplicate wells. **P < 0.01, ***P < 0.001, NS indicating not significant (Student’s *t*-text). Abbreviation: SjHSP60, *S. japonicum* heat shock protein 60.

To determine whether the SjHSP60 antibody level increases with *S. japonicum* infection, C57BL/6 J mice were infected with 12 *S. japonicum* cercariae. Eight weeks after infection, the SjHSP60 antibody levels in the sera from experimentally infected mice were measured by ELISA. The results revealed that the SjHSP60 IgG level was significantly increased in the sera from the infected mice compared with that from the controls. The subtype IgG1 exhibited the same tendency as the total IgG, whereas the level of the IgG2a subtype exhibited no significant difference between the two groups (Fig. 1A).

To better illustrate whether the SjHSP60 antibody titers were correlated with the severity of liver pathology in the acute stage, C57BL/6 J mice were infected with 12, 20, or 40 *S. japonicum* cercariae. The liver granuloma sizes, based on histology, in the mice were determined 8 weeks after infection. The results revealed that both SjHSP60 IgG (Fig. 1B) and the subtype IgG1 (Fig. 1C) antibody levels were positively correlated with the sizes of the hepatic granulomas in the *S. japonicum*-infected mice (R = 0.755, P < 0.001 and R = 0.615, P < 0.001, respectively). Additionally, the SjHSP60 IgG and IgG1 antibody levels were strongly increased five weeks after infection, which paralleled the initial formation of the granulomas in the mouse livers (Fig. 1D–F).

### Correlations between the SjHSP60 antibody levels and the severities of liver pathology in the acute and chronic stages in rabbits

**Figure 2:**
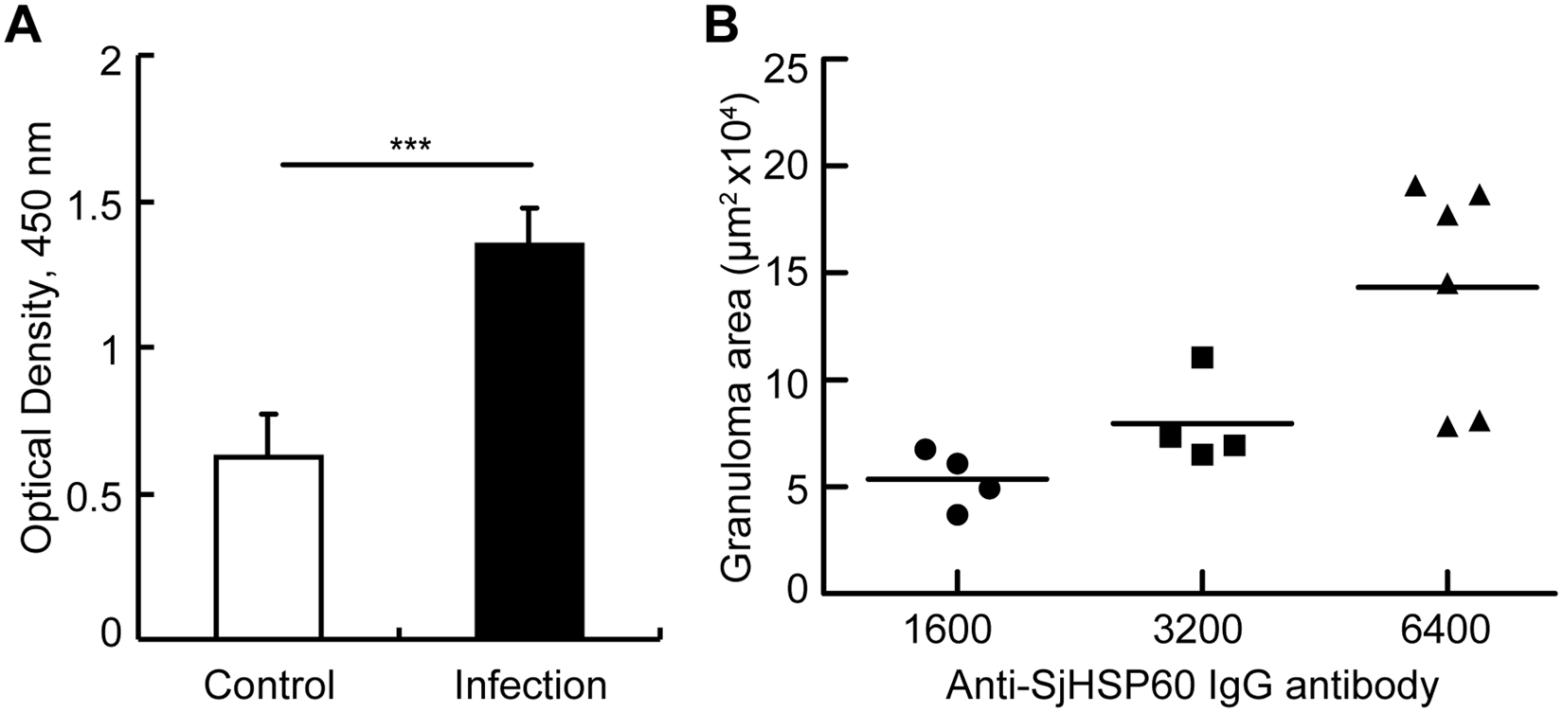
Correlation between the levels of SjHSP60 antibodies and the sizes of the hepatic granulomas in the *S. japonicum*-infected rabbits 8 weeks after infection. (**A**) Rabbits were infected with or without 60 *S. japonicum* cercariae. Eight weeks later, the sera were collected, and anti-SjHSP60 total IgG antibody was determined by ELISA in triplicates. The results are presented as the means ± the SDs from six rabbits in each group and are representative of 3 independent experiments. ***P < 0.001 (Student’s *t*-test). (**B**) Sera and liver slices were obtained from rabbits infected with 30 or 60 *S. japonicum* cercariae. The associations of the titers of SjHSP60 IgG antibody with the sizes of the granulomas in *S. japonicum*-infected rabbits were determined by Spearman’s rank correlation. The sizes of the granulomas around single eggs were quantified with the AxioVision Rel 4.7. The data are expressed in area units (n = 14). Anti-SjHSP60 total IgG antibody was determined by ELISA. The data are expressed in serum dilution (1/x). The data are representative of 3 independent experiments. Abbreviation: SjHSP60, *S. japonicum* heat shock protein 60.

Schistosomiasis is likely a chronic and low-grade infective disease^2, 21^, and hepatic fibrosis significantly accumulates during the chronic stage. We assessed a possible link between the levels of SjHSP60 antibody and the progression of hepatic granuloma and fibrosis. Rabbits were infected with or without 30 or 60 *S. japonicum* cercariae. Eight weeks later, the serum SjHSP60 antibody levels (Fig. 2A) and the granuloma sizes were measured (Fig. 2B). Consistent with the results from the infected mice, the results presented in Fig. 2 demonstrated significantly elevated levels of SjHSP60 IgG antibodies in the infected rabbits (Fig. 2A) and a positive correlation between SjHSP60 IgG antibody level and granuloma size (R = 0.764, P < 0.001) in the liver at week 8 after infection (Fig. 2B).

Since type I collagen (collagen I), type β collagen (collagen III), and alpha-smooth muscle actin (α-SMA) have been linked to the progression of hepatic fibrosis^22–25^, we further examined whether these proteins were also correlated with the levels of SjHSP60 IgG antibodies. Twenty-three weeks after infection, the levels of SjHSP60 IgG antibodies and the progression of hepatic fibrosis were measured (Table 1). The result revealed that the amounts of collagen III (R = 0.596, P < 0.001) and α-SMA (R = 0.678, P < 0.001) were significantly correlated with the levels of SjHSP60 IgG antibodies (Table 1). Together, these results confirmed a positive correlation between the SjHSP60 IgG antibody level and the severity of liver pathology in *S. japonicum*-infected rabbits.

**Table 1:** Collagen III and α-SMA, but not collagen I levels of expression were significantly associated with the level of serum SjHSP60 IgG antibody.

Titer of SjHSP60 IgG antibody	Amounts of expressions of proteins	Collagen I			collagen III			α-SMA		
		Cases (n = 58)			Cases (n = 58)			Cases (n = 58)		
		I	II	III	I	II	III	I	II	III
200		2	1	3	6	0	0	5	1	0
400		2	3	2	5	2	0	4	3	0
800		7	8	3	9	9	0	8	9	1
1600		5	4	6	5	9	1	3	8	4
3200		5	3	4	0	10	2	0	2	10

The sera and liver slices were from rabbits at 23 weeks after infection with 30 or 60 *S. japonicum* cercariae. The levels of expressions of collagen I, collagen III, and α-SMA in the total livers were evaluated by a skilled pathologist. The anti-SjHSP60 total IgG antibody was determined by ELISA. The data are expressed in serum dilution (1/x). The data were combined from 3 independent experiments and analyzed. In this experiments, we observed that collagen III (r = 0.596, P < 0.001) and α-SMA (r = 0.678, P < 0.001), but not collagen I, levels of expression were significantly associated with the serum level of SjHSP60 IgG antibody. The correlations were analyzed using Spearman’s rank correlation.

Abbreviation: SjHSP60, *S. japonicum* heat shock protein 60.

### Correlation between the SjHSP60 antibody level and the severity of hepatic fibrosis in schistosomiasis patients

**Figure 3:**
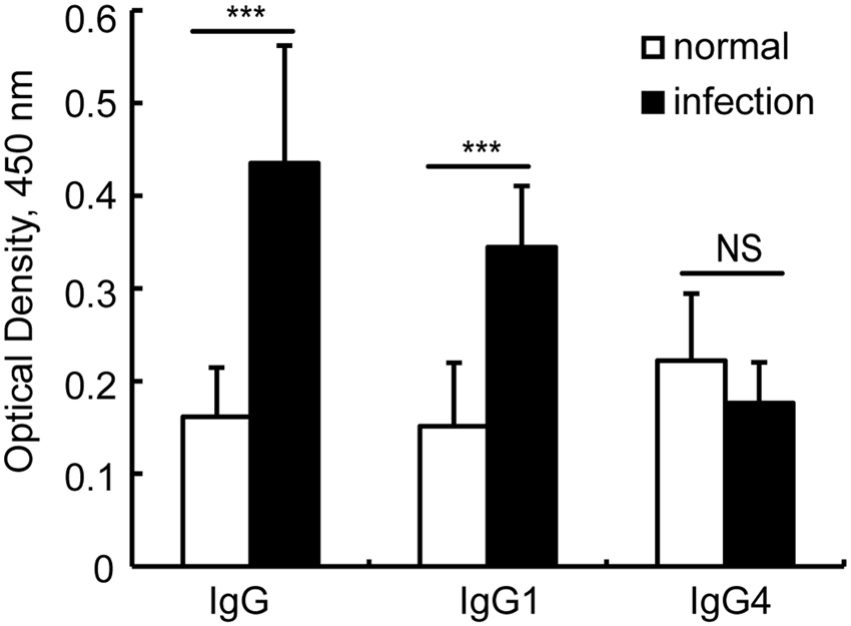
High levels of SjHSP60 total IgG and subtype IgG1 antibodies in *S. japonicum*-infected patients. Sera were collected from normal participants (n = 10) and the age- and sex-matched patients selected from serum storage (n = 10). Anti-SjHSP60 total IgG, IgG1 and IgG4 antibodies were determined by ELISA in triplicate wells. The bar represents the mean ± SD. ***P < 0.001, NS indicating not significant (Mann-Whitney *U* test). Abbreviation: SjHSP60, *S. japonicum* heat shock protein 60.

In human schistosomiasis, the progression of liver pathology is the primary cause of chronic morbidity and mortality^26, 27^. To further confirm whether there is also a correlation between SjHSP60 antibody level and the severity of hepatic fibrosis in schistosomiasis patients, a total of 61 patients and 10 healthy controls were recruited. There was no statistically significant difference in the age distributions of the patients and healthy controls. However, there was a significant difference in the gender distributions of the patients and healthy controls (Table 2). Age- and sex-matched patients (n = 10) were selected for comparison with the healthy controls to study whether the SjHSP60 antibody levels were elevated in the patients. In accordance with the findings from the infected animals, we found that the levels of SjHSP60 total IgG and subtype IgG1, but not IgG4, were significantly higher in the patients (Fig. 3). Importantly, there were significantly positive correlation of the levels of serum SjHSP60 total IgG (R = 0.614, P < 0.01) and IgG1 (R = 0.368, P < 0.001) antibodies with the severity of hepatic fibrosis in the patients (Table 3). However, no significant correlation of SjHSP60 IgG4 with liver pathology was observed in the patients with schistosomiasis japonica (see Supplementary Table S1).

**Table 2:** The demographic and clinical characteristics of subjects.

**Parameters**		**HC**	**Schistosomiasis Japonica**		**P-value**
Number		10	61		
Age (years)					
Mean ± SD	37.6 ± 10.16 (24–55)			43.89 ± 19.35 (12–84)	>0.05 (Mann-Whitney *U* test)
Sex N (%)					
Male	5 (50%)		38 (62.3%)		<0.05 (Pearson Chi-Square Test)
Female	5 (50%)		23 (37.7%)		

HC, healthy control.

**Table 3:** Correlations of the titers against SjHSP60 antibody with the severity of liver pathology in *S. japonicum*-infected patients.

Severity of liver pathology	Titer of SjHSP60 IgG1 antibody							Titer of SjHSP60 IgG antibody						
	Cases (n = 61)							Cases (n = 61)						
	50	100	200	400	800	1600	3200	800	1600	3200	6400	12800	25600	51200
I	2	5	3	7	10	5	0	2	5	6	15	4	0	0
II	0	0	6	5	3	5	2	0	1	4	3	5	5	3
III	0	0	0	0	2	5	1	0	0	0	0	2	5	1

Sera were collected from *S. japonicum*-infected patients (n = 61). Anti-SjHSP60 total IgG and IgG1 antibodies were determined by ELISA. The data are expressed in serum dilution (1/x). Liver fibrosis was evaluated by ultrasound using the WHO grading scale. There were associations of the SjHSP60 IgG and IgG1 antibody levels with the severity of liver pathology in the infected patients (for SjHSP60 IgG r = 0.614, P = 0.004; IgG1 r = 0.368, P < 0.001). The correlations were analyzed using Spearman’s rank correlations.

Abbreviation: SjHSP60, *S. japonicum* heat shock protein 60.

### High SjHSP60 total IgG antibody titer in *S. japonicum*-infected mice treated with CCl_4_

**Figure 4:**
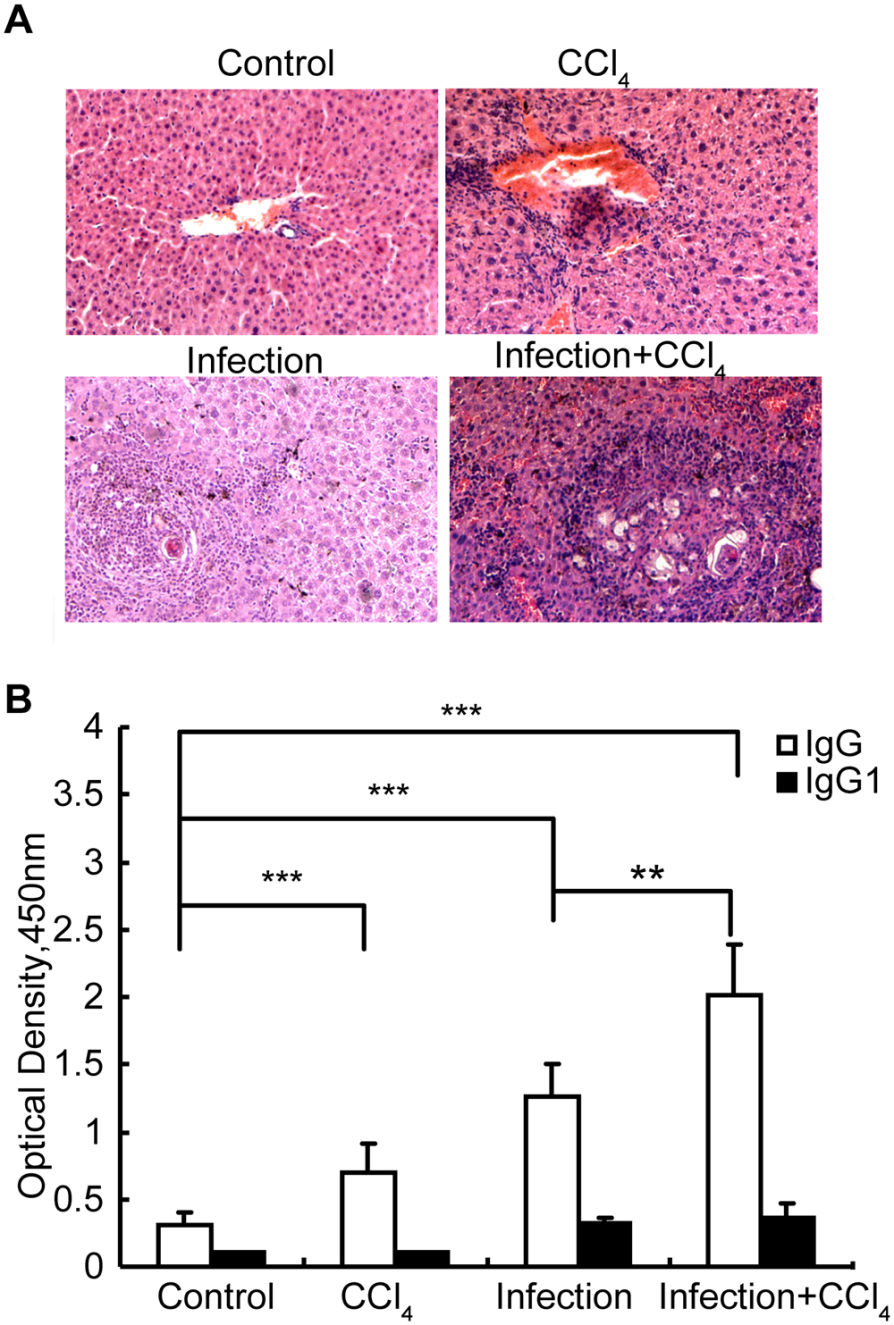
Higher titer of SjHSP60 total IgG antibody in infected mice treated with CCl_4_. Normal or *S. japonicum*-infected mice with or without CCl_4_ treatment were sacrificed at 8 weeks after infection. (**A**) Representative histology liver sections were stained with H&E to reveal granulomas. The results are shown at × 200 original magnification. (**B**) The sera anti-SjHSP60 total IgG and IgG1 antibody levels were determined by ELISA in triplicate wells. The error bars indicate the means ± the SDs from six mice in each group. **P < 0.01, ***P < 0.001 (Student’s *t*-text). The data are representative of 3 independent experiments. Abbreviation: SjHSP60, *S. japonicum* heat shock protein 60.

It is commonly known that some *S. japonicum*-infected patients are likely to suffer from comprehensive liver damage with exposure to not only *S. japonicum* infection but also other adverse factors, such as long-term alcohol abuse, drug treatment and coinfections with hepatitis viruses^28^. To evaluate whether the levels of SjHSP60 antibodies could be used to monitor the severity of liver pathology in cases of such comprehensive liver damage, we treated *S. japonicum*-infected mice with CCl_4_ as an inducer of comprehensive liver damage according to the methods of Novobrantseva *et al*.^29^. We found a significant exacerbation of liver injury in *S. japonicum*-infected mice treated with CCl_4_ compared with the *S. japonicum*-infected mice without CCl_4_ treatment (Fig. 4A)_._ Moreover, a significantly higher level of the SjHSP60 IgG, but not the IgG1 antibody, was also observed in infected mice after treatment with CCl_4_ (Fig. 4B), which suggests that the level of SjHSP60 IgG is suitable for evaluating combined liver damage.

Therefore, our results suggest that SjHSP60 total IgG antibody is a promising biomarker for liver pathology in schistosomiasis.

## Discussion

In this study, we illustrated that the level of SjHSP60 antibody was significantly correlated with the severity of liver pathology in hosts with schistosomiasis japonica and reported the SjHSP60 antibody as a promising biomarker to screen for schistosomiasis japonica and monitor liver pathological progression during infection.

The severity of liver pathology in schistosomiasis has been demonstrated to vary with not only the egg load in host liver but also with immunologic differences between hosts^30, 31^. Previous studies have demonstrated that the severity of pathology resulting from immune responses is significantly correlated with the level of antibody in some specific diseases rather than only with the amount of antigen^32–36^. Similarly, our data revealed that the levels of SjHSP60 antibody were associated with the severity of liver pathology after schistosome infection, which strongly suggests that SjHSP60 antibody is a promising biomarker for evaluating liver pathology in schistosomiasis japonica hosts. However, the possible molecular foundation for this correlation remains unknown and to be investigated.

Previous research into schistosomiasis, especially in animal models, is generally highly reductionist, i.e., it focuses on the disease-causing agent while meticulously excluding extraneous factors. However, the real world is quite different; there can be multiple concurrent hepatitis viruses, bacteria, and alcohol abuse, and each has differing dynamics and influences on the host liver. Indeed, some patients with schistosomiasis are also influenced by coinfections, such as hepatitis B and C infections^37, 38^, or have suffered from other influential liver injury factors, such as prolonged alcohol abuse-induced liver degeneration, nonalcohol-induced steatohepatitis and/or long term use of some drugs that induce liver damage. The hallmarks of the above liver damage are chronic inflammation, cellular damage, regeneration, and fibrosis, which can be evoked by repeated CCl_4_ injection^29^. Therefore, we treated mice with CCl_4_ to mimic the co-pathogenesis of schistosomiasis with these concurrent agents in the liver. Our data suggest that the level of SjHSP60 antibody is also correlated with the severity of the liver pathology in *S. japonicum*-infected mice treated with CCl_4_. Heat shock proteins (HSPs) are a group of molecular chaperones that are highly conserved from prokaryotes to higher eukaryotes^39^, and our unpublished data indicate that the mammalian homologues of SjHSP60 share approximately 70% and 72% sequence similarity with the sequences of mice and humans, respectively. It is thus plausible that the increased level of SjHSP60 antibody in *S. japonicum*-infected mice treated with CCl_4_ may be due to cross-reaction between SjHSP60 with increased HSP60 from host liver cells that have been damaged due to concurrent liver injury factors.

Evaluation of liver pathology is important not only for implementing appropriate therapeutic interventions for liver pathology in schistosomiasis patients but also for epidemiological research. Because schistosomiasis japonica is endemic in poor and undeveloped countries or areas, there is a great need for physicians to mass-screen patients in a relatively reliable, cheap, and less-device dependent manner. Thus, our study suggests that the reflection of the overall severity of liver damage, including other influencing factors, by the level of SjHSP60 IgG antibody makes this level a promising biomarker for repeatedly mass-screening and monitoring the pathological progression of overall liver damage in schistosomiasis japonica patients in endemic areas.

In conclusion, our study identified SjHSP60 antibodies as candidate biomarkers for monitoring liver pathology in defined schistosomiasis patients, which is not only helpful for identifying the proper interventions for liver pathologies in individual patients with schistosomiasis japonica but also suitable for repeatedly mass-screening patients to monitor the progression of liver pathology in epidemiological studies of schistosomiasis japonica. However, validation of these potential biomarkers needs to be investigated in our further studies.

## Methods

### Animals and Infection

Eight-week-old male C57BL/6 J mice and rabbits (Helminth-naive, specific pathogen-free, New Zealand white, male, 2.2–2.4 kg) were purchased from SLAC Laboratory (Shanghai, China). Cercariae of *S. japonicum* (Chinese mainland strain) was routinely maintained in *Oncomelania hupensis* snails purchased from the Jiangsu Institute of Parasitic Diseases (Wuxi, China) and obtained by exposing infected snails to light for 1–2 h to induce shedding of the cercariae. The number of cercariae and their viability were determined using a light microscope. Each mouse or rabbit was percutaneously infected with *S. japonicum* by placing a glass slide carrying a series of different numbers of cercariae (12, 20, or 40 cercariae for the mice, and 30 or 60 for the rabbits) on abdomen for 20 minutes to form differential pathological levels of liver granuloma or fibrosis. The mice were sacrificed 3, 5, or 8 weeks after infection to investigate the correlations of SjHSP60 antibodies with the size of the liver granuloma. The rabbits were sacrificed at 8 or 23 weeks after infection to investigate the associations of SjHSP60 antibodies with the size of the liver granuloma or the severity of liver fibrosis, respectively^40^. The combined liver damage in the *S. japonicum*-infected mice was induced by intraperitoneal (i.p.) injection with 2 ml/kg body weight of 10% CCl_4_ (Sigma-Aldrich, St. Louis, MO) dissolved in olive oil (Sigma-Aldrich) three times a week for 8 weeks^41^. One day after the first injection, the mice were infected with *S. japonicum* cercariae as described above. Mice that were not exposed to *S. japonicum* infection or CCL_4_ treatment were used as controls. The animal experiments were performed in strict accordance with the Regulations for the Administration of Affairs Concerning Experimental Animals (1988.11.1), and all efforts were made to minimize suffering. All animal procedures were approved by the Institutional Animal Care and Use Committee (IACUC) of Nanjing Medical University (Permit Number: NJMU 09-0163). The methods were performed in accordance with the relevant guidelines and regulations

### Patients

All human study subjects were from a village in Jiujiang City, Jiangxi Province in a *S. japonicum* low-transmission area of the southeastern Poyang Lake region. The inhabitants became infected with *S. japonicum* through frequent water contact in the snail-infested marshlands close to the village due to agricultural activities and fishing. The subjects included 10 healthy adult controls and 61 patients with schistosomiasis japonica as confirmed by egg detection using the Kato-Katz method with duplicate examinations of 3 consecutive stool specimens obtained from each individual^42^. However, the schistosomiasis patients and healthy controls, whose stool examination results were positive for other parasite eggs, such as *Ascaris lumbricoides* and *Trichuris trichiura*, were excluded from our study while performing the stool examinations. The individuals with positive stool examination results for schistosome eggs were treated with a single oral dose of praziquantel (40 mg/kg). The healthy controls did not display histories of or laboratory or clinical signs of schistosome infection, did not suffer from coinfections with HBV or HCV, and did not use medication two weeks before blood collection. Ethical clearance for this study was obtained from the Institutional Review Board of Nanjing Medical University, Nanjing, China (Permit Number: 2009NMUIEC101). All human-related methods were performed in accordance with the relevant guidelines and regulations. Written informed consent was obtained from each subject.

### Ultrasound Evaluation

Hepatic fibrosis was evaluated by ultrasound using the WHO grading scale ^43^. All examinations were evaluated by two trained ultrasonographers using a single portable ultrasound machine with 3.5 MHz probe (Hitachi Corporation, Tokyo, Japan) with the participants in the supine position. Both ultrasonographers were blinded to the infection status. Liver ultrasonography was conducted according to the 1990 draft guidelines^44, 45^. Liver parenchymal fibrosis was graded 1 through 3 based on observed lesions or 0 if none was present. Periportal fibrosis was assessed by grading the average outer wall to outer wall diameter of three peripheral branches of the portal vein between the first and third branching point (grade 0: 3 mm; grade 1: 3 to 5 mm; grade 2: 5 to 7 mm; grade 3: 7 mm). The internal diameter of the portal vein was measured at the entry point of the portal vein into the liver. The length of the left liver lobe was measured in a longitudinal section along the left parasternal line, and the length of the right liver lobe was measured as the maximum oblique diameter using a right anterior axillary view according to the revised guidelines^43^.

### Expression and purification of SjHSP60

The pBluescript-SKII-SjHSP60 plasmid containing the full-length cDNA of SjHSP60 was previously constructed^46^. The SjHSP60 coding region of approximately 1.7 kb was PCR amplified using a 5′ end primer (5′-cgcggatcccaaccggtgacaatgttacgag-3′) with a *Bam*HI site and a 3′ end primer (5′-ccgctcgagattaagagcaggcagtgtttac-3′) with an *Xho*I site. The full-length HSP60 coding region was cloned into the pGEX-6P-1 expression vector (Amersham Bioscience, Piscataway, NJ) at the *Bam*HI and *Xho*I restriction sites and then transformed into the Escherichia coli strain BL21 (Novagen, Madison, WI). After confirmation by DNA sequencing, expression of the GST-SjHSP60 fusion protein was induced with 0.1 mM isopropyl-1-thio-β-D-galactopyranoside (IPTG) for 5 h at 37 °C, and the fusion protein was purified with glutathione-Sepharose 4B (GE Healthcare, Piscataway, NJ) affinity chromatography. Recombinant SjHSP60 was then released by cleaving with PreScission Protease (GE Healthcare) according to the manufacturer’s specifications. The purity of the recombinant SjHSP60 (>95%) was confirmed by sodium dodecyl sulfate-polyacrylamide gel electrophoresis (SDS-PAGE) followed by Coomassie Blue staining. Additionally, Polymyxin B-Agarose (Sigma-Aldrich, St. Louis, MO) was used to remove LPS and LPS-associated molecules in the recombinant SjHSP60 preparations. The endotoxin activity (<0.001 FU/ml, 0.1 pg/ml) was determined using the LAL assay kit (BioWhittaker, Walkersville, MD) according to the manufacturer’s instructions.

### Antibody assays

The serum levels SjHSP60 IgG, IgG1, IgG2a and IgG4 antibodies were measured by ELISA following previously described methods^47^. Briefly, each well of a 96-well plate (Costar, Cambridge, MA) was coated with 100 ng recombinant SjHSP60 protein, mouse HSP60 protein (Enzo Life Sciences, Plymouth Meeting, PA) or OVA in 100 μL coating buffer overnight at 4 °C. The plate wells were washed three times in 0.05% Tween 20 (PBS-T) and blocked for 2 h with 5% milk powder in PBS-T at 37 °C. The test sera were added, and the plates were incubated overnight at 4 °C for maximal sensitivity. The plate wells were washed three times, and 100 μl/well of detection antibody (HRP-Goat Anti-Human IgG, HRP-Mouse Anti-human IgG1, HRP-Mouse Anti-human IgG4, Southern Biotech, Birmingham, AL; HRP- Rat Anti-mouse IgG1, HRP- Rat Anti-mouse IgG, HRP- Rat Anti-mouse IgG2a, HRP-Goat anti-Rabbit IgG (H + L), BD Pharmingen, San Diego, CA) diluted in PBS-T was added. The plates were sealed and incubated for 1 h at 37 °C. The plate wells were washed 5 times with PBS-T. The residual buffer was removed from the plates on absorbent paper, and then 100 μl/well of TMB (eBioscience, San Diego, CA) was added to each well. The plates were incubated at room temperature for 15 min, and 50 μl of Stop Solution (2 mol/L H_2_SO_4_) was added to each well. The plates were read at 450 nm.

### Pathology assessment

The livers from the mice and rabbits were fixed in 10% neutral buffered formalin. 4-μm paraffin-embedded sections were dewaxed and stained with hematoxylin and eosin (H&E). For each mouse or rabbit, the sizes of 30 granulomas around single eggs were quantified with the AxioVision Rel 4.7 (Carl Zeiss GmbH, Jena, Germany). The data are expressed in area units. To determine the severity of the hepatic fibrosis, morphometric analysis of type I collagen (Novus Biologicals, Littleton, CO), type III collagen (Acris Antibodies GmbH, Herford, Germany) and α-SMA (Abcam, Cambridge, MA) accumulation were performed at 100× magnification using an Axiovert 200 M microscope. A skilled, blinded pathologist evaluated the percentages of hepatic collagen in the liver sections. For semiquantitative analysis of the expressions of type I collagen, type III collagen, and α-SMA, all liver sections of each individual mouse were scored as I, II, or III (I for < 25%, II for 25–50%, III for > 50% of each field occupied by the staining-positive area), and an average score was then calculated for each individual mouse to represent its live pathology status.

### Statistical analysis

All data were analyzed using SPSS 11.0 software. The optical densities (OD) are presented as the mean ± the SD and were analyzed using Student’s *t* test or Mann-Whitney *U* tests. SjHSP60 IgG, IgG1 or IgG4 antibody titers were compared between groups with different pathologies using Spearman’s rank correlation. The differences at p < 0.05 were considered statistically significant.

## Electronic supplementary material

Supplementary Table S1
