# Supplementary material for: Elevated serum antibody against Schistosoma japonicum HSP60 as a promising biomarker for liver pathology in schistosomiasis
Source: Sci Rep. 2017 Aug 10;7:7765. doi: 10.1038/s41598-017-08283-5 (PMC5552731; doi:10.1038/s41598-017-08283-5)
Supplement: Supplementary file 1 — Supplementary Table S1 [file 41598_2017_8283_MOESM1_ESM.pdf]

Elevated serum antibody against *Schistosoma japonicum* HSP60 as a promising biomarker for liver pathology in schistosomiasis

Xiaojun Chen[1], Wei Li[1], Yalin Li[1], Lei Xu[1], Sha Zhou[1], Jifeng Zhu[1], Zhipeng Xu[1,*], Feng Liu[1], Dandan Lin[2], Fei Hu[2], Yuemin Liu[2], Wen Jiang[3], Liwang Cui[4], Chuan Su[1,*]

Supplementary Information

Supplementary Table S1 Correlation of the titer against the SjHSP60 IgG4 antibody with the severity of liver pathology in *S. japonicum*-infected patients

Severity of liver pathology	Titer of SjHSP60 IgG4 antibody Cases (n=61)					
	5	10	20	40	80	160
I	8	8	5	8	3	0
II	4	5	1	7	4	0
III	5	0	0	0	2	1

Sera were collected from *S. japonicum*-infected patients (n=61). The anti-SjHSP60 IgG4 antibody was determined by ELISA. The data are expressed in serum dilutions (1/x). Liver fibrosis was evaluated by ultrasound using the WHO grading scale. No significant correlation of SjHSP60 IgG4 with liver pathology was observed in patients with schistosomiasis japonica ($r=0.038$, $P=0.769$). The correlations were analyzed using Spearman's rank correlation.

Abbreviation: SjHSP60, *S. japonicum* heat shock protein 60.
